# The ANeED study – ambroxol in new and early dementia with Lewy bodies (DLB): protocol for a phase IIa multicentre, randomised, double-blinded and placebo-controlled trial

**DOI:** 10.3389/fnagi.2023.1163184

**Published:** 2023-05-26

**Authors:** Luiza Jadwiga Chwiszczuk, Monica Haraldseid Breitve, Bjørn-Eivind Bordewick Kirsebom, Per Selnes, John Chr. Fløvig, Anne-Brita Knapskog, Ragnhild E. Skogseth, Jessica Hubbers, Elin Holst-Larsen, Arvid Rongve

**Affiliations:** ^1^Department of Old Age Related Medicine, Haugesund Hospital, Helse Fonna Trust, Haugesund, Norway; ^2^Department of Research and Innovation, Haugesund Hospital, Helse Fonna Trust, Haugesund, Norway; ^3^Department of Clinical Neuropsychology, Haugesund Hospital, Helse Fonna Trust, Haugesund, Norway; ^4^Department of Neurology, University Hospital of North Norway, Tromsø, Norway; ^5^Department of Neurology, Akershus University Hospital, Lørenskog, Norway; ^6^Sant Olavs Hospital, Trondheim, Norway; ^7^Department of Geriatric Medicine, Oslo University Hospital, Oslo, Norway; ^8^Department of Internal Medicine, Haraldsplass Deaconess Hospital, Bergen, Norway; ^9^Senior Clinical Research Associate in ProToCall, Haugesund, Norway; ^10^Institute of Clinical Medicine, University in Bergen, Bergen, Norway

**Keywords:** DLB, dementia with Lewy bodies (DLB), ambroxol, treatment, clinical trial in DLB, alpha-synuclein

## Abstract

**Background:**

Currently, there are no disease-modifying pharmacological treatment options for dementia with Lewy bodies (DLB). The hallmark of DLB is pathological alpha-synuclein (aS) deposition. There are growing amounts of data suggesting that reduced aS clearance is caused by failure in endolysosomal and authophagic pathways, as well as and glucocerebrosidase (GCase) dysfunction and mutations in the GCase gene (GBA). The population’s studies demonstrated that the incidence of GBA mutations is higher among Parkinson’s disease (PD) patients, and carriers of such mutations have a higher risk of developing PD. The incidence of GBA mutations is even higher in DLB and a genome-wide association study (GWAS) confirmed the correlation between GBA mutations and DLB. *In vivo* experiments have shown that ambroxol (ABX) may increase GCase activity and GCase levels and therefore enhance aS autophagy-lysosome degradation pathways. Moreover, there is an emerging hypothesis that ABX may have an effect as a DLB modifying drug. The aims of the study “Ambroxol in new and early Dementia with Lewy Bodies (ANeED) are to investigate the tolerability, safety and effects of ABX in patients with DLB.

**Methods:**

This is a multicentre, phase IIa, double-blinded, randomised and placebo-controlled clinical trial, using a parallel arm design for 18 months’ follow-up. The allocation ratio is 1:1 (treatment:placebo).

**Discussion:**

The ANeED study is an ongoing clinical drug trial with ABX. The unique, but not fully understood mechanism of ABX on the enhancement of lysosomal aS clearance may be promising as a possible modifying treatment in DLB.

**Trial Registration:**

The clinical trial is registered in the international trials register – clinicaltrials.com (NCT0458825) and nationally at the Current Research Information System in Norway (CRISTIN 2235504).

## Background

In our memory clinics in Western Norway, approximately one in five patients is recognized to have dementia receive the diagnosis of dementia with Lewy bodies (DLB) – a disease associated with a more rapid decline, earlier nursing home admission and death, higher caregiver burden and higher levels of behavioural and psychiatric symptoms (BPSD) than in Alzheimer’s disease (AD) (Rongve et al., 2013). After Robin Williams’ suicide due to severe DLB, awareness of the disease rose, increasing disease recognition and priority. The DLB Association established research priorities with a focus on creating infrastructure for clinical trials and more resources to develop new therapeutics in 2019 (Peterson et al., 2019). Currently, there is no disease-modifying treatment for DLB (Lee et al., 2019).

Among the challenges associated with clinical trials in DLB are lack of standardised clinical assessment tools, lack of established and standardised biological markers, heterogeneity of DLB symptomatology and disease course and, importantly, difficulties regarding early identification of the disease (Goldman et al., 2020).

Lessons learned from many unsuccessful clinical trials on symptomatic AD have led to the conclusion that disease modifying treatment should be initiated as soon as possible, ideally in the pre-clinical stages (Cummings et al., 2018). Early interventions are required before extensive, evident neuronal damage such that the effectivity of pharmacological treatment reaches its full potential, and patients in prodromal stages must therefore be included in clinical trials. Over time, the recognition of the prodromal stages of DLB has increased, including subjective cognitive (SCD) and mild cognitive impairment (MCI). For the first time, diagnostic research criteria were recently published (McKeith et al., 2017). As clinical symptoms are even less specific and less pronounced in the prodromal stage, sensitive and specific biomarkers are crucial for timely and accurate diagnosis of prodromal DLB and for prediction of conversion towards DLB. Because of the unfulfilled promises of newly developed drugs for AD and the sustained lack of a new pharmacological strategy in DLB, researchers have focused on drug repurposing or repositioning in neurodegenerative disorders.

The hypothesis that ambroxol (ABX) could potentially be useful in DLB originated in studies on Gaucher disease (GD), a lysosomal storage disorder, caused by a mutation in the glucocerebrosidase (GCase) gene (*GBA1*) and resulting in the development of parkinsonism in some patients (Tayebi et al., 2003). This has led researchers to discover that mutations in *GBA1* are a genetic risk factor for synucleinopathies similar to Parkinson’s disease (PD) and DLB (Goker-Alpan et al., 2004; Lwin et al., 2004). Between 2 to 30% of patients with PD have *GBA1* mutations, increasing the risk of PD up to 20–30 fold (Neumann et al., 2009). The frequency is even higher in DLB, particularly in Ashkenazi Jews (Donaghy et al., 2018). One of the studies demonstrated a significant association between *GBA1* mutation carrier status and other synucleinopathies such as DLB. Subjects with DLB were more than 8 times more likely to carry a *GBA1* mutation than controls (Nalls et al., 2013). The mechanisms by which *GBA1* mutations resulted in Parkisonian symptoms in GD have been unclear until researchers found a link between GCase activity and alpha-synuclein (aS). GCase deficiency causes pathologic accumulation of aS (Velayati et al., 2010; Cullen et al., 2011), while GCase overexpression in the brain or gene therapy may reduce pathology and memory deficits in a synucleinopathy mouse model (Sardi et al., 2011). PD and DLB patients without GCase mutation also exhibit lower levels of GCase activity in the central nervous system (CNS), and this is related to earlier disease onset and worse cognitive and non-motor symptoms (Gegg et al., 2012; Chiasserini et al., 2015). This suggests a potential contribution of the GCase activity in disease pathogenesis, possibly by alteration of lysosomal function. Modulation of the enzyme’s activity might increase the lysosomal autophagic clearance of aS (Papadopoulos et al., 2018).

Unfortunately, current treatment for patients with GCase deficiency by enzyme replacement or gene therapy is not sufficient or successful in all patients. This is why researchers have begun to search for alternative treatment avenues. Some of them target the proteasome and autophagy dysfunction and include chaperone-mediated autophagy (CMA) and macroautophagy (Webb et al., 2003; Vogiatzi et al., 2008; Wildburger et al., 2020). Such drugs were designed to bind and modify aS misfolding and promote lysosomal transport by improving GCase activity (Elkouzi et al., 2013; Bonam et al., 2021).

Nowadays, there is extensive research on the use of pharmacological chaperones to enhance lysosomal GCase activity, such as ABX, isofagomine or NCGC607 (Han et al., 2020).

ABX, a drug used to treat airway mucus hypersecretion, has shown effects in both animal models and human GD type 3 (GD3) with predominant neurological symptoms and PD (McNeill et al., 2014; Richards et al., 2016). Preclinical trials of ABX in models of synucleinopathies are promising, incising the effects of GCase and reducing the concentration of toxic aS species. For an overview of pre-clinical animal studies, see the supplementary materials. Here, we describe human studies.

The pilot study of five patients with GD3 and a positive *in vitro* chaperone test showed that high-dose ABX treatment had good safety and tolerability, significantly increased lymphocyte GCase activity, permeated the blood–brain barrier and decreased glucosylsphingosine levels in the cerebrospinal fluid, with motor improvement in two of them (Narita et al., 2016). Additionally, an over four-year-long study on the safety and efficacy of combined high-dose ABX (up to 27 mg/kg/day) and enzyme replacement therapy in four patients with GD3 and myoclonic epilepsy showed that seizure frequency significantly decreased from baseline, neurocognitive function improved and the drug was tolerated without severe adverse events (AEs) (Kim et al., 2020).

The first study on PD was a non-randomised, non-controlled trial phase IIa trial of 17 participants with mutation in *GBA1* and without. The results showed that ABX penetrated to the CNS, had a modulatory effect on GCase in cerebrospinal fluid (CSF) and increased total aS concentration in CSF. The researchers observed a tendency towards mild improvement in the total score of the motor part of Unified Parkinson1s disease Rating Scale (UPDRS), but the lack of a placebo group made it difficult to interpret the findings. The treatment was safe and well tolerated (Mullin et al., 2020). The second study on Parkinson’s disease dementia (PDD) randomised, placebo controlled and quadruple masked with 75 participants started in 2015 (Silveira et al., 2019). The study completed inclusions and follow-ups, and preliminary results showed that ABX has been well tolerated and that there were no severe AEs judged to be due to ABX. The researchers also reported that GCase levels in white blood cells increased by 1.6-fold (oral report on the AD/PD conference, Barcelona 2022).

There are other ongoing or planned studies on ABX in DLB. One of them is a placebo-controlled study of 15 participants with DLB, which will last 52 weeks (Stephen Pasternak, Lawson Health Research Institute, London, Ontario, Canada). The estimated study completion is September 2024 (NCT04405596). The same centre is responsible for the study “Ambroxol as a Treatment for Parkinson’s Disease Dementia” in 75 individuals with PDD. The researchers estimated study completion to December 2021 (NCT02914366), but the results are as of yet not published. The researchers from Israel started the register for patients with GD and GBA carriers with PD (300 participants). The goal of this register is to complete data within 10 years and further use this as a reference for patients, caregivers and physicians who consider the use of ABX (REGISTRY for the Collection of Real World Data on the Safety and Efficacy of Ambroxol – World Data on Ambroxol for Patients with GD and GBA Related PD; Ari Zimran, Shaare Zedek Medical Centre [NCT04388969]). For a better overview of clinical trials with ABX, see Table 1.

**Table 1:** Clinical studies on ambroxol in synucleinopathies.

Study/type	Dgn.	Status	Dosing	N	Duration	Main outcome
Ambroxol as a Treatment for Parkinson’s Disease Dementia, London, Canada	PDD	Ongoing, NCT02914366	1,050 mg oral	55	52 weeks	ADAS-cog
Ambroxol in Disease Modification in Parkinson Disease (AIM-PD), London, United Kingdom	PD	Completed	1,260 mg oral	20	6 months	Safety, tolerability and pharmacodynamics
Ambroxol as a Novel Disease Modifying Treatment for Lewy Body Dementia, London, Canada	DLB	Not yet recruiting, NCT04405596	1,350 mg oral	15	52 weeks	MMSE
Ambroxol as a disease-modifying treatment in GBA-PD (AMBITIOUS), multicenter, Italy	PD	Ongoing, NCT05287503	1,200 mg oral	60	52 weeks	MoCA
Ambroxol in New and Early DLB, A Phase IIa Multicenter Randomized Controlled Double Blind Clinical Trial (The ANeED Study), multicenter, Norway	DLB	Ongoing, NCT04588285	1,260 mg	172	18 months +12 months open extension	MMSE

ADAS-cog, the Alzheimer’s Disease Assessment Scale-Cognitive Subscale; DLB, Dementia with Lewy bodies; MMSE, the Mini Mental State Examination; MoCA, the Montreal Cognitive Assessement; PD, Parkinson’s disease; PDD, Parkinson’s disease dementia.

The main objectives of our study are: 1) to assess the safety and tolerability of ABX at high dosage in DLB patients, 2) to examine the effect of ABX on the rate of cognitive decline and associated motor and non-motor DLB symptoms, 3) to study the effect of ABX on dementia markers in CSF, neuroimaging and neurophysiological markers (resting state EEG [rsEEG]), and 4) to study the rate of functional decline. If the drug tolerance is good, we hope that this study will provide new insights that can lead towards a new standard treatment for DLB.

### Methods

The team at Helse Fonna, Haugesund Hospital, Department of Research and Innovation is the sponsor and we designed the study protocol based on the published AIM-PD protocol (Silveira et al., 2019). The funding agency is the public and government organisation, KLINBEFORSK, the national program for clinical treatment research in the specialist health services.

### Design

This is a multicentre, phase IIa, double blinded, randomised and placebo-controlled clinical trial, using a parallel arm design for 18 months’ follow up. The allocation ratio is 1:1 (treatment:placebo). The participants will undergo detailed clinical assessments at the screening visit, baseline visit (day 1) and at weeks 4, 8, 24, 36, and 52 of the treatment phase. We will perform four phone controls in the escalation phase and at week 5, 12, 16, 20, 28, 32, 40, 44, and 48. Between screening visit and baseline (max. 90 days), we will perform mandatory lumbar punctures, MRI, DaTSCAN, ECG, rsEEG, blood analysis, clinical and cognitive assessments.

Each participant will receive five intra-participant dose escalations at 60 mg three times a day (TID) (days 1–7), 120 mg TID (days 8–14), 315 mg twice a day (BID) (days 15–28), 315 mg TID (days 22–28) and 420 mg TID (days 29–550) with ABX or placebo for the duration of 18 months. After this period, we will offer the treatment with ABX in an open-label extension phase for 12 months for all participants who will continue.

The study is carried out in seven Norwegian centres: Helse Fonna, Haugesund Hospital as the study-coordinating center and Haraldsplass Deaconess Hospital (Bergen), Stavanger University Hospital, Akershus University Hospital (Oslo), Oslo University Hospital, St. Olav Hospital (Trondheim) and the University Hospital of North Norway (Tromsø) are allocated study sites. The inclusion period began in May 2021.

### Participants

Participants must fulfil the following inclusion criteria: a) diagnosis of possible or probable DLB or MCI-DLB according to the established McKeith criteria from 2017 and 2020, respectively (McKeith et al., 2017; IG et al., 2020), b) are in age range between 50 and 85 years, c) have MMSE score > 15 at screening, d) are on stable doses of antiparkinsonian or dementia or psychiatric medications at least 1 month prior to the study initiation, e) participant or participant’s caregiver are able to give informed consent to actual and further assessments and procedures and f) have a responsible caregiver >18 years old who has contact with the participant at least three times a week.

The main exclusion criteria are a) any clinically significant or unstable psychiatric, medical or surgical condition, b) major cardiovascular or cerebral event that occurred within 6 months prior to the first visit, c) advanced malign disease or other terminal illness, d) relevant medication, alcohol or drug abuse, e) known sensibility to or contraindication for ABX, f) female participants who are pregnant or currently breastfeeding, and g) not able to proceed with the study regime (i.e., like traveling to study site or dysphagia, or unable to perform supplementary examination because of contraindications, claustrophobia, etc.). For other detailed inclusion and exclusion, criteria see Table 2.

**Table 2:** Inclusion and exclusion criteria in the ANeED study.

Inclusion criteria	1. Age ≥ 50 and ≤ 85 years of age, both gender
	2. Confirmed diagnosis of DLB including mild cognitive impairment in DLB (DLB-MCI).
	3. MMSE score >=15 at screening
	4. Able and willing to provide informed consent prior to any study related assessments and procedures
	5. Capable of complying with all study procedures
	6. Willing to provide blood samples for genetic analyses of APOE and GBA
	7. Willing and able to self-administer or administer by a caregiver oral ambroxol medication, from day 1 to study end (at 60 mg TID (day 1-7), 120 mg TID (day 8- 14), 315 mg BID (day 15-21), 315 mg TID (day 22-28) and 420 mg TID (day 29-550)).
	8. Contact with caregiver at least 3 times a week, to ensure sufficient information from the caregiver regarding participant’s status and possible change in condition
	9. Able to travel to the participating study site
	10. A female participant is eligible to participate if she is of non-childbearing potential or if women is of childbearing potential must use accepted contraceptive methods
	11. Caregiver needs to be ≥ 18 years when signing the informed consent form
Exclusion criteria	1. Current treatment with anticoagulants (e.g. warfarin, argatroban, dabigatraneteksilat, rivaroksaban, apiksaban, edoksaban)
	2. Current use of IMP or participation in another interventional clinical trial
	3. Exposure to more than three IMP within 12 months prior to the first dose in the current study
	4. Confirmed dysphagia that would preclude self-administration of ambroxol up to six tablets/d for the duration of this study
	5. Known sensitivity to the study medication, ambroxol or its excipients (lactose monohydrate, granulated microcrystalline cellulose, silicon dioxide, magnesium stearate and denatonium benzoate)
	6. History of known rare hereditary disorders of galactose intolerance: lactase deficiency or glucose-galactose malabsorption
	7. History of severe substance abuse (drug abuse or alcohol)
	8. Donation of blood (1 unit or 350 ml) within three months prior to receiving the first dose of the study drug
	9. Pregnant or breastfeeding
	10. Any clinically significant or unstable medical or surgical condition that may put the participant at risk when participating or may influence the results of the study or affect the participant’s ability to take part in the study. Such conditions may include:
	a) impaired renal function defined by eGFR<=30
	b) moderate/severe hepatic impairment defined by Child-Pugh score >1
	c) a major cardiovascular event (e.g. myocardial infarction, acute coronary syndrome, decompensated congestive heart failure, pulmonary embolism, coronary revascularisation that occurred within 6 months prior to the screening visit)
	d) Major stroke
	e) Major depression defined clinically or by GDS-15 >=11 points or delirium or psychotic disorder unrelated to DLB.
	f) Cancer, history of metastatic cancer, terminal illness or clinically significant disease within ≤5 years, except for adequately treated basal cell skin cancer.
	11. Planned major surgical treatment during the study period

APOE, apolipoprotein epsilon; BID, twice a day; DLB, dementia with Lewy bodies; DLB-MCI, mild cognitive impairment in DLB; eGFR, estimated glomerular filtration rate; GDS, the Geriatric Depression Scale; IMP – investigational medicinal product, PD, Parkinson’s disease, PDD, dementia in PD; MMSE, Mini Mental State Examination; TID, three times a day.

If the patient at a later time point is no longer eligible to participate in the study as a result of a change in health status that does not align with the inclusion or exclusion criteria, the principal investigator (PI) will decide if the deviation will result in exclusion from the scheduled follow-ups. Minor deviations, such as a recently developed depression or cognitive decline on the Mini Mental State Examination (MMSE) <15, provide no solid base for exclusion. Major deviations, such as a change in diagnosis DLB (e.g., to non-DLB) may result in exclusion from the study.

To assess informal caregivers’ stress, we wish to invite them to join the study. Informal caregivers to join the study as well. Informal caregivers are eligible to join if they are spending time with the patient at least three times a week.

### Recruitment

We plan to recruit participants primarily through specialists or delegated trained clinicians working in the study’s sites: memory outpatient’s clinics, geriatrics clinics, and old age psychiatry clinics or during routine standard of care outpatient appointments at participating sites. The PI or delegated clinician in each of the participating centres will clinically evaluate whether participants fulfil all inclusion criteria without any counterindications. Haugesund Hospital is the appointed participant identification centre (PIC) and refers all potential participants to their local site after national patient and public involvement (PPI) initiatives. Additionally, all sites will identify their own local participants.

As an additional recruitment alternative, we will apply for ethical approval to search medical records for patients attending our sites during the last 12 months to be contacted for screening. If the projected study sample size turns out to be low, we will also engage the PPI program, which will focus on patient recruitment through various media channels (paper, TV and other advertisements).

### Randomisation and blinding

We will perform randomisation using the electronic case report form (eCRF) system (Viedoc^1^). The participants will be randomly assigned into two arms: ABX 1260 mg/d (in three doses per day) and placebo – in an allocation ratio of 1:1. Before randomisation, the participants will be stratified due to risk factors (number of APOEε4 alleles (0, 1, 2) and beta-amyloid in CSF: normal and low). Therefore, we will use a restricted randomisation method with blocking and stratification. The number of strata is six. The block size is 4 (McKeith et al., 2017) with number and blocks 6 (Webb et al., 2003). The independent data manager will provide randomisation, and the drug manufacturer will get the list and deliver drug kits numbered based on the stratification.

The patients, patient’s caregivers, all study personnel, monitors and statisticians are blinded until the end of the study, with the exception of an emergency unblinding procedure. The ABX and placebo are produced in a tablet form and identical in appearance and taste to blinding purposes. Placebo tablets with microcrystalline cellulose as the main compound will include denatonium benzoate as a residual amount of bitter substance as ABX tablets have. Each participant will get the investigation medical product (IMP) in sequentially numbered identical containers according to the randomisation schedule. The responsible pharmaceutical company, Kragerø Tablettproduksjon AS, will send IMP shipments directly to each participating centre for dispensing and storage for the study.

### Interventions and assessment procedures

If a recruited participant fulfils the eligibility criteria, the inclusion procedure begins with a screening visit. During the screening visit, the PI or a delegated clinician verifies the eligibility criteria and evaluates the competency of the patient, willingness and ability to comply with the protocol. If the participant and informal caregiver give written consent, the patient is formally included in further assessment.

The screening visit includes medical history, standard physical examination (vital signs, weight, height, orthostatic blood pressure and optionally smell test), neurological examination with focus on extrapyramidal symptoms (UPDRS-III), and ECG. During the visit, we also perform also extended cognitive examination (including MMSE 3rd Norwegian revised Edition) (MMSE-NR3, five different sub-versions to minimize the training effect) (Folstein et al., 1975), the clock test (CT), the Consortium to Establish a Registry for Alzheimer’s Disease (CERAD) word list test (Fillenbaum et al., 2008), the Visual Object and Space Perception (VOSP) battery subtest silhouettes (Warrington and James, 1991), Trail Making Test A and B (TMT A and B) (Reitan and Wolfson, 1985) and the Controlled Oral Word Association Test (COWAT) phonemic fluency FAS test (COWAT-FAS) (City, 1986). We also screen psychiatric and dementia symptoms using standardised tools, such as the Geriatric Depression Scale (GDS) for depression and the Clinical Dementia Rating-Sum of Boxes (CDR-SB) for staging of dementia. Caregiver interviews includes the Mayo Sleep Questionnaire (MSQ) to assess sleep disturbances, the Mayo Fluctuations Scale (MFS) to assess fluctuations, the Neuropsychiatric Inventory (NPI) for neuropsychiatric symptoms, the Relative Stress Scale (RSS) and the Informant Questionnaire on Cognitive Decline in the Elderly.

The next steps are to perform obligatory examinations (MRI cerebrum, DaTSCAN/CIT-SPECT, lumbar puncture, rsEEG and blood taking) within a maximum of 90-day period from the screening day. After a maximum of 90 days after the screening, we will perform the baseline visit and participants will receive the first dose of IMP under strict monitoring.

From this point forward, the first 18-months phase treatment regime begins, and it is divided into the titrations phase and the maintenance phase. Initially, each participant will receive five intra-participant IMP dose escalations at 60 mg TID (baseline – day 7), next 120 mg TID (days 8–14), 315 mg BID (days 15–28), 315 mg TID (days 22–28), and 420 mg TID, a total of 1,260 mg/d. To track the side effects tied to the study drug, all patients will receive a weekly phone call in the dose-escalation phase to monitor safety and tolerability. Changes in medication use (other than the study drug), any AEs or falls will also be assessed during these phone calls. In the case of side effects, the PI can decide to deviate from the drug admission protocol in order to adjust or delay dose escalation.

**Figure 1:**
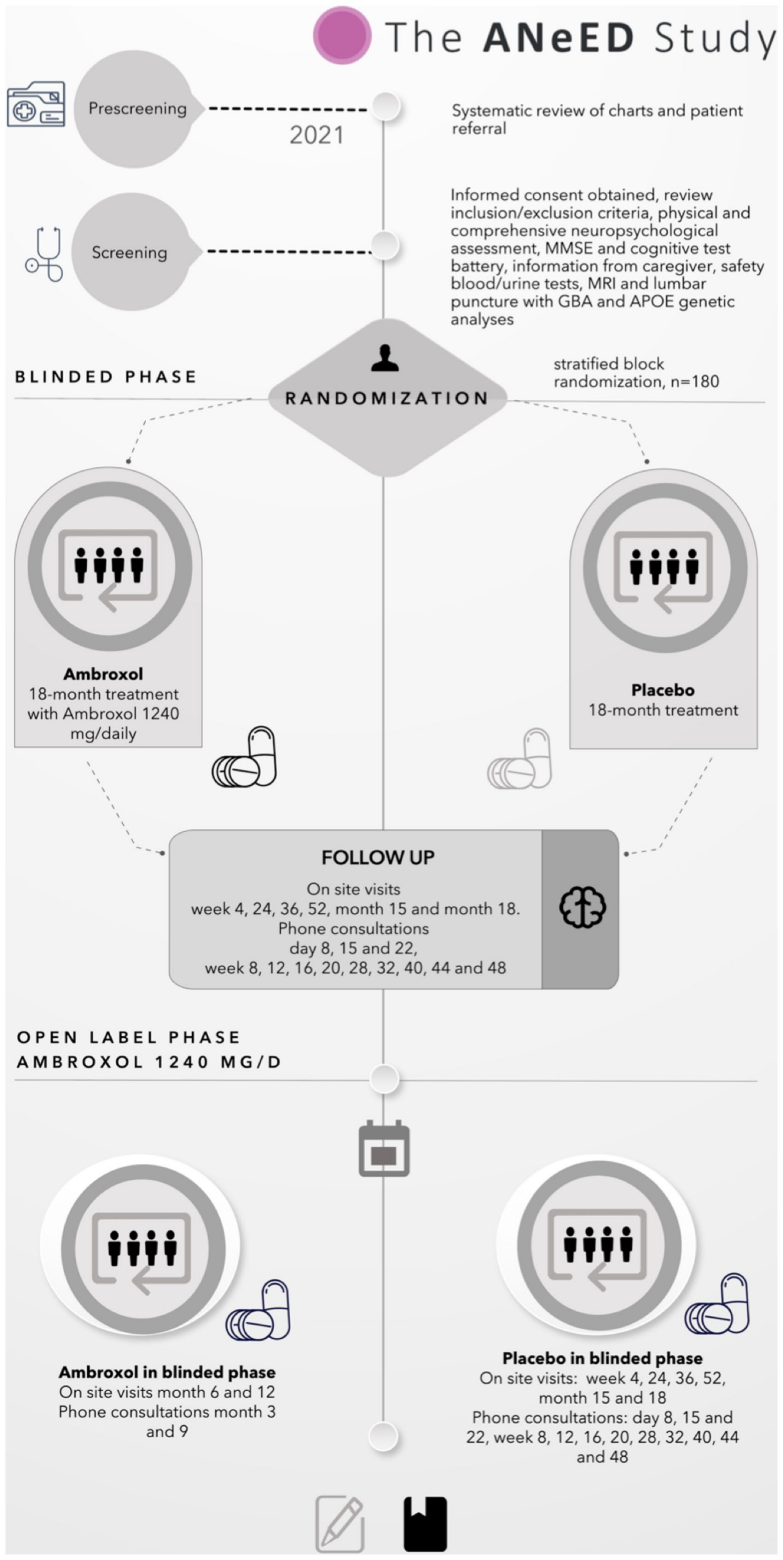
The flow chart of the ANeED study’s schedule.

All participants will have eight scheduled on-site visits: an initial screening, a drug administration visit, 6 follow-up visits, and a total 16 phone calls spread out over 18 months. Figure 1 shows the flow chart of the ANeED study’s schedule.

### Safety monitoring

During the follow-up visits, the physical examination, vital signs, ECG, MDS-UPDRS, neurological examination and laboratory measures will be repeated. We will monitor and register all AEs since the last visit. The clinical investigator (CI) will be responsible for reviewing each AEs, estimating its severity and possibility of relationship to IMP and finally deciding whether this is an indication to cease medication. We will register and report all AEs, regardless of their relation to trial treatment.

In life-treating conditions, the PI in each centre will handle suspicions of possible severe interactions and requests from the other clinician responsible for the patient.

### Primary outcome

The primary outcome is the mean score on the MMSE-N3 at 18 months in the intervention group compared to the control group. Secondary outcomes are as follows:

1. To establish safety and tolerability of the ABX in high doses. The endpoints are the number of participants withdrawing the study medication in ABX and placebo groups, the number of participants requiring reduction of dosage due to possible AE in ABX and placebo groups and the number and characterisations of AEs in both study arms.
2. To establish the effect of ABX in subgroups of participants diagnosed with DLB measured by MMSE-NR3 in ABX and placebo groups dependent on stratified risk for concomitant beta-amyloid pathology defined by *APOE* status and beta-amyloid (Aβ_1-42_) levels in CSF.
3. To assess the effect of ABX on the results of neuropsychological tests (change from baseline to 18 months in mean scores for the clock drawing test, COWAT immediate and delayed recall, TMT A and B, VOSP silhouettes and FAS test).
4. To study the effect of ABX on the global functional decline measured by changes in CDR-SB at the baseline and after 18 months.
5. To investigate the effect of the ABX on neuropsychiatric symptoms and depression measured by the mean change in the total NPI score and GDS, respectively, from baseline to the study end at month 18.
6. To examine the effect of ABX on sleep disturbances, especially REM sleep behaviour disorders (RBD), frequency of falls, cognitive and awareness fluctuations, parkinsonism and autonomic functions. Endpoints are change in the number of participants with RBD defined from the MSQ, the frequency of falls at the baseline and study end, the change in the MFS and the mean change in the UPDRS motor part score at the baseline to month 18 in ABX and placebo arms.

### Ancillary studies

We will also perform a number of optional sub-studies:

1. Neurophysiological study using data from rsEEG registration at the baseline and at the end of the trial. This method is non-invasive and relatively cheap for detecting early changes in cerebral activity and may prove to be a viable marker for distinguishing DLB from AD. We aim to characterise rsEEG parameters in patients with DLB in comparison with AD and healthy age matched controls from the European DLB Consortium EEG database.
2. Neuroimaging study based on MRI data. Cerebral MRI anatomical neuroimaging scans without contrast (3D T1, T2 (TSE + 3D FLAIR), DWI and SWI) will be carried out on either 1.5 or 3 T scanners depending on site.
We will perform volumetric measurement and multi-modal and post-processing techniques to characterise patterns of cerebral pathology in the early stages of DLB. Additionally, it will be of interest to examine putative changes in these parameters before and after treatment with ABX.
3. CSF fluid and blood biomarker studies. These will be planned to determine if treatment with ABX potentially has an effect on brain amyloidosis (Aβ_1-42_), tau-tangles (p-tau), and neurodegeneration (t-tau). To assess aS levels in CSF we chose CSF Real-Time Quaking-Induced conversion method. The genotyping will be performed for the presence of *GBA1* mutations and to determine APOE allele status. The GCase levels in serum will be taken, before treatment and at the end of treatment period.
4. Remote disease and treatment monitoring study. As soon as possible, we will implement high-tech digital methods, such as smartphones, smart glasses, smart headbands and non-contact smart sleep monitor (Somnofy) to register the spectrum of vital signs, sleep parameters, motor activity and simplified EEG and eye tracking data. Additionally, we aim to implement real-time gait or walking activity monitoring to assess motor symptoms in DLB and changes under treatment.
5. Caregivers’ stress study. Caregivers’ engagement is an integral part of a patient’s care but may often be connected with emotional and mental burnout. We wish to assess the caregivers’ stress levels and to find out what critical moments in patient care lead to distress. Such an analysis will be a starting point for developing of improvement procedures.

### Sample size

The primary outcome in this study is the mean score between the two groups (placebo and ABX) at 18 months. Both groups included patients with prodromal DLB and DLB. Based on our published data from the Dementia Study of Western Norway, where we included persons living with mild DLB (MMSE 23 points average at baseline) with annual follow-up, we found that the annual decline during the first 2 years of follow-up is 2 points for DLB with a standard deviation of 5.7. Based on these data, we estimated a 3-point decline at 18 months. We applied a sample power analysis to calculate the number of participants needed to detect a 3-point difference in the MMSE-NR3 scores at 18 months for 80% power to find a difference between the active treatment group and the placebo group and accounted for 25% drop-out. Based on these data, we estimated the sufficient number of participants to be 180.

### Statistical methods

Full details of all planned statistical analyses will be included in a written statistical analysis plan (SAP), which will be completed before the final study database is locked for analysis. The SAP will specify those endpoints and will employ conventional frequentist methods (i.e., statistical significance tests). All final reports and publications will clearly identify and differentiate between pre-planned and post-hoc tests of statistical significance. When appropriate, value of p thresholds will be adjusted to compensate for increases in family-wise error rates associated with multiple testing. We will apply an ANCOVA, a linear model for MMSE at 18 months, depending on treatment adjusted for MMSE at baseline with a random intercept for randomisation stratification and block.

### Data handling and storage

The data will be handled in accordance with the European Union (EU) General Data Protection Regulation and in accordance to the GCP. The eCRFs will not bear the participants’ names or other personal identifiable data. The participants’ trial identification numbers will be used for identification and this will be clearly explained to the participants on the patient’s information sheet.

All study sites have access to the digital eCRF platform Viedoc (see text footnote 1). This platform is assigned to manage health and research data and has been approved for drug trials in the EU/European Economic Area including Norway. Each participant receives a personal identification number to ensure that the data are anonymised. Additionally, all CRFs are stored in paper forms and are safely archived. All other data in electronic forms (EEG, MRI and DaTSCAN) are archived in local, secure servers held by the Information and Communication Technologies service in Norway.

All study personnel are trained to comply with GCP regulations, protocols, and all standard operating procedures (SOPs). Additionally, all CRFs are stored in paper forms and are safely archived.

Archiving will be authorised by the sponsor following submission of the end of the study report in collaboration with Viedoc. The CI is responsible for the secure archiving of essential trial documents and the trial database as per their trust policy. All essential documents will be archived for a minimum of 15 years after completion of the trial and in adherence to the Norwegian Medicine Agency’s (Statens Legemiddelverk) recommendations.

Frequent meetings will be held between and across the study sites in order to ensure quality and consistency. The data will be frequently monitored on site, and including but not limited to source data verification, inclusion and exclusion criteria (serious), AEs protocol deviations and IMP drug data.

## Discussion

Here, we present here our protocol for the ANeED study testing ABX in prodromal and mild DLB. Prodromal stages of DLB are now defined for research purposes and these individuals, including those with mild dementia and those who need to be offered inclusion in clinical trials to establish treatment options with disease modifying drugs in DLB.

The rationale for the use of ABX in DLB is firmly established and is based on both preclinical and clinical data. Because GCase activity is associated with aS metabolism, enhancing CMA may potentially reduce pathological aS depositions. Preclinical trials of ABX in models of synucleinopathies are promising and showing the effects of reducing the concentration of toxic aS species. ABX penetrate into CSF (Mullin et al., 2020) and first trials have shown an enhancing effect of ABX on GCase activity in human cells.

Our study is the largest ongoing clinical trial with ABX and the first in patients with prodromal and mild DLB. To date, we have included 47 patients in the study and 30 who started medication in the first 12 months; we observed good tolerability with only a few side effects. There are also seven candidates waiting for a screening visit.

One previous study on ABX in PD was carried out in only 17 participants, and it was not randomised or placebo controlled (Mullin et al., 2020); another is now completed with 50 patients with PDD (Silveira et al., 2019). The goal in our study is to recruit 180 participants, a number sufficient to find a difference between the active treatment group and the placebo group at 18 months based on the MMSE. However, 180 participants might not be enough to apply for a marketing authorisation if the study turns out to show the effect of ABX on DLB.

Based on our previous research, we know that the combined significant AD pathology in DLB will worsen prognosis; therefore, we decided to stratify the participants in our study based on APOEε4 genotypes and CSF Aβ_1-42_ to secure equal groups for placebo and ABX. We will also extend the study duration to 18 months and thereafter to 12 months of open extension to maximise the chances of showing treatment effects.

In this study, we will include an extensive biomarker program including MRI, rsEEG, genetics and a broad spectrum of digital bio-markers. We will also implement the extremely promising CSF Real-Time Quaking-Induced Conversion method for assessing aS-pathology in CSF. This method has a reported sensitivity above 95%, with a specificity of 98% for DLB and other synucleinopathies (Rossi et al., 2020). We hope that our study will bring more knowledge about ABX’s safety, tolerability and efficacy in the drug treatment of synucleinopathies, such as DLB. We also hope that treatment will show effects on both clinical outcome measures, and digital and traditional CSF biomarkers.

### Ethics

The study protocol and all informed consent forms were approved by the Regional Ethics Committee (REK 73910) and other regulatory organs, such as the Norwegian Medicine Agency and Norwegian Centre for Research Data (NSD; 582,472), prior to any participants’ recruitment. The trial is registered in the international trials register - www.clinicaltrials.gov (NCT0458825) and nationally at the Current Research Information System in Norway (CRISTIN 2235504). It is also the responsibility of the CI, PI or designee at each site to ensure that all subsequent amendments gain the necessary approvals, including approval from data protection official at the site. This does not affect the individual clinician’s responsibility to take the immediate action necessary to protect the health and interests of individual participants for reporting urgent safety measures.

All participants, or – if participant due to the mental disability, a participant will not be able to give consent – caregivers, will be informed about the study, study procedures and medication and will have the right to discuss all doubts and questions before enrolment. The participants will be free to refuse to participate in any study phase and for any reason. They will be asked whether they wish to be told the results of their genotyping and counselled accordingly as part of standard of care procedures by the local PI or study clinician.

## Ethics statement

The studies involving human participants were reviewed and approved by Regional Ethics Committee, REK 73910. The patients/participants provided their written informed consent to participate in this study.

## Author contributions

LC was responsible for conception of the manuscript, wrote the main manuscript text, prepared figures and tables, and made substantial contributions to the conception. LC, MB, and JH drafted the work and all the other authors substantively revised it. EH-L and AR were responsible for clinical trial design and protocol. LC, MB, B-EK, PS, JF, A-BK, RS, JH, EH-L, and AR contribute in data acquisition under the study duration, designed the work, and reviewed the manuscript. All authors contributed to the article and approved the submitted version.

## Funding

The work was supported by KLINBEFOSK, 2019201.

## Conflict of interest

The authors declare that the research was conducted in the absence of any commercial or financial relationships that could be construed as a potential conflict of interest.

## Publisher’s note

All claims expressed in this article are solely those of the authors and do not necessarily represent those of their affiliated organizations, or those of the publisher, the editors and the reviewers. Any product that may be evaluated in this article, or claim that may be made by its manufacturer, is not guaranteed or endorsed by the publisher.

## Footnotes

^1^ https://www.viedoc.com
