# Supplementary material for: The ANeED study – ambroxol in new and early dementia with Lewy bodies (DLB): protocol for a phase IIa multicentre, randomised, double-blinded and placebo-controlled trial
Source: Front Aging Neurosci. 2023 May 26;15:1163184. doi: 10.3389/fnagi.2023.1163184 (PMC10250712; doi:10.3389/fnagi.2023.1163184)
Supplement: Supplementary file 1 [file Table_1.DOCX]

Pre-clinical studies on ambroxol in synucleinopathies

| **Study/paper/reference** | **Author**  **year** | **Conclusion** |
| --- | --- | --- |
| Identification and characterization of ambroxol as an enzyme enhancement agent for GD (1) | Maegawa  2009 | ABX has the biochemical characteristics of a safe and effective enzyme enhancement agent for the treatment of patients with GD patients. |
| The chaperone activity and toxicity of ambroxol on Gaucher cells and normal mice (2) | Luan  2013 | ABX significantly increased the GCase activity in the spleen, heart and cerebellum of the mice. Good oral availability and wide distribution and chaperone activity in the tissues, including the brain and its lack of acute toxicity. |
| Ambroxol as a pharmacological chaperone for mutant glucocerebrosidase (3) | Bendikov Bar  2013 | ABX increases the lysosomal fraction and the enzymatic activity of several mutant GCase variants in skin fibroblasts derived from Type 1 and Type 2 GD patients. |
| Ambroxol improves lysosomal biochemistry in glucocerebrosidase mutation-linked Parkinson disease cells (4) | McNeill  2014 | Treatment with ABX increased GCase activity in fibroblasts from healthy controls, GD and heterozygous GCase mutation carriers with and without PD. ABX significantly increases GCase activity and reduces markers of oxidative stress in cells bearing GCase mutations. |
| Ambroxol-induced rescue of defective glucocerebrosidase is associated with increased LIMP-2 and saposin C levels in GBA1 mutant Parkinson's disease cells (5) | Ambrosi  2015 | ABX increased cathepsin D, GCase activity and Sap C protein levels in all groups, while LIMP-2 levels were increased only in GBA1-mutant PD fibroblasts. |
| Ambroxol effects in GCase and α-synuclein transgenic mice (6) | Migdalska-Richards  2016 | ABX treatment resulted in increased brain GCase activity in (1) wild-type mice, (2) transgenic mice expressing the heterozygous mutation in the murine GCase 1 gene, and (3) transgenic mice overexpressing human alpha-synuclein. |
| Oral ambroxol increases brain glucocerebrosidase activity in a nonhuman primate (7) | Migdalska-Richards  2017 | ABX treatment increased GCase activity in cynomolgus monkeys. 20% increase in GCase activity in the midbrain, cortex, and striatum. ABX might be capable of crossing the primate brain‐blood barrier and increasing wild‐type GCase activity. |
| Ambroxol modulates 6-Hydroxydopamine-induced temporal reduction in glucocerebrosidase enzymatic activity and PD symptoms (8) | Mishra  2018 | The results suggest that Ambroxol attenuated 6-OHDA-induced GCase deficiency and PD symptoms. |
| Effects of ambroxol on the autophagy-lysosome pathway and mitochondria in primary cortical neurons (9) | Magalhaes, 2018 | Mitochondria content increased by ABX via peroxisome PGC1-α. Study suggest that ABX, besides being a GCase chaperone, also acts on other pathways (mitochondria, lysosomal biogenesis and the secretory). |
| Drosophila melanogaster mutated in its GBA1b ortholog recapitulates neuronopathic GD (10) | Cabaso  2019 | Reduced unfolded protein response. Reduced neuroinflammation. Enhanced lifespan. |
| Neurorestorative effects of sub-chronic administration of ambroxol in rodent model of Parkinson's disease (11) | Mishra  2020 | ABX restored TH and DAT levels and extracellular DA concentration, indicating the recovery of dopaminergic system. GCase enzymatic and mitochondrial complex-I activity were restored, and aS pathology was decreased |
| Ambroxol increases GCase activity and restores GCase translocation in primary patient-derived (12) macrophages in GD and parkinsonism Kopytova:2021cc} | Kopytova, 2021 | GD macrophage treatment resulted in increased GCase level and increased enzyme colocalization with the lysosomal marker LAMP2. |
| Ambroxol reverses tau and α-synuclein accumulation in a cholinergic N370S GBA1 mutation model (13) | Yang, 2022 | ABX significantly enhanced GCase activity and decreased both tau and aS levels in cholinergic neurons. |

Abbreviations: 6-OHDA – 6 – hydroxydopamine; aS – alpha synuclein, ABX – ambroxol, GD – Gaucher disease, GCase – glucocerebrosidase, GBA1 – glukocerebrosidase gene, LIMP2 - lysosomal integral membrane protein-2; LAMP2 - lysosomal-associated membrane protein 2A; PD- Parkinson’s disease, Sap C – saposin C , PGC1-alpha –proliferator-activated receptor gamma coactivator, TH – tyrosine hydroxylase

References:

1. Maegawa GHB, Tropak MB, Buttner JD, Rigat BA, Fuller M, Pandit D, et al. Identification and characterization of ambroxol as an enzyme enhancement agent for Gaucher disease. J Biol Chem. American Society for Biochemistry and Molecular Biology; 2009 Aug 28;284(35):23502–16.

2. Luan Z, Li L, Higaki K, Nanba E, Suzuki Y, Ohno K. The chaperone activity and toxicity of ambroxol on Gaucher cells and normal mice. Brain and Development. 2013 Apr;35(4):317–22.

3. Bendikov-Bar I, Maor G, Filocamo M, Horowitz M. Ambroxol as a pharmacological chaperone for mutant glucocerebrosidase. Blood Cells Mol Dis. 2013 Feb;50(2):141–5.

4. McNeill A, Magalhaes J, Shen C, Chau K-Y, Hughes D, Mehta A, et al. Ambroxol improves lysosomal biochemistry in glucocerebrosidase mutation-linked Parkinson disease cells. Brain. 2014 May;137(Pt 5):1481–95.

5. Ambrosi G, Ghezzi C, Zangaglia R, Levandis G, Pacchetti C, Blandini F. Ambroxol-induced rescue of defective glucocerebrosidase is associated with increased LIMP-2 and saposin C levels in GBA1 mutant Parkinson's disease cells. Neurobiol Dis. 2015 Oct;82:235–42.

6. Richards AM, Daly L, Bezard E, Schapira AHV. Ambroxol effects in glucocerebrosidase and α‐synuclein transgenic mice. Ann Neurol. John Wiley & Sons, Ltd; 2016 Nov 1;80(5):766–75.

7. Migdalska-Richards A, Ko WKD, Li Q, Bezard E, Schapira AHV. Oral ambroxol increases brain glucocerebrosidase activity in a nonhuman primate. Synapse. John Wiley & Sons, Ltd; 2017 Jul;71(7):e21967.

8. Mishra A, Chandravanshi LP, Trigun SK, Krishnamurthy S. Ambroxol modulates 6-Hydroxydopamine-induced temporal reduction in Glucocerebrosidase (GCase) enzymatic activity and Parkinson's disease symptoms. Biochem Pharmacol. 2018 Sep;155:479–93.

9. Magalhaes J, Gegg ME, Migdalska-Richards A, Schapira AH. Effects of ambroxol on the autophagy-lysosome pathway and mitochondria in primary cortical neurons. Sci Rep. Nature Publishing Group; 2018 Jan 23;8(1):1–12.

10. Cabasso O, Paul S, Dorot O, Maor G, Krivoruk O, Pasmanik-Chor M, et al. Drosophila melanogaster Mutated in its GBA1b Ortholog Recapitulates Neuronopathic Gaucher Disease. Journal of Clinical Medicine 2022, Vol 11, Page 3809. Multidisciplinary Digital Publishing Institute; 2019 Sep 9;8(9):1420.

11. Mishra A, Krishnamurthy S. Neurorestorative effects of sub-chronic administration of ambroxol in rodent model of Parkinson’s disease. Naunyn-Schmiedeberg's Arch Pharmacol. Springer Berlin Heidelberg; 2020 Mar 1;393(3):429–44.

12. Kopytova AE, Rychkov GN, Nikolaev MA, Baydakova GV, Cheblokov AA, Senkevich KA, et al. Ambroxol increases glucocerebrosidase (GCase) activity and restores GCase translocation in primary patient-derived macrophages in Gaucher disease and Parkinsonism. Parkinsonism Relat Disord. 2021 Mar;84:112–21.

13. Yang SY, Taanman J-W, Gegg M, Schapira AH. Ambroxol reverses tau and α-synuclein accumulation in a cholinergic N370S GBA1 mutation model. Human Molecular Genetics (2022) (In press). Oxford University Press (OUP); 2022 Feb 18.
